# The spreading of the invasive sacred ibis in Italy

**DOI:** 10.1038/s41598-020-79137-w

**Published:** 2021-01-08

**Authors:** Marco Cucco, Gianfranco Alessandria, Marta Bissacco, Franco Carpegna, Mauro Fasola, Alessandra Gagliardi, Laura Gola, Stefano Volponi, Irene Pellegrino

**Affiliations:** 1 University of Piemonte Orientale, DISIT, viale Michel 11, 15100 Alessandria, Italy; 2 GPSO, Museo di Storia Naturale, 12022 Carmagnola, TO Italy; 3 University of Pavia, DEES, via Ferrata 6, 27100 Pavia, Italy; 4 University of Insubria, DISTA, via Dunant 3, 21100 Varese, Italy; 5 Parco del Po, piazza Giovanni XXIII 6, 15048 Valenza, AL Italy; 6 ISPRA, via Ca’ Fornacetta 9, 40064 Ozzano Emilia, BO Italy

**Keywords:** Zoology, Invasive species, Population dynamics

## Abstract

The spreading of invasive species in new continents can vary from slow and limited diffusion to fast colonisations over vast new areas. We studied the sacred ibis *Threskiornis aethiopicus* along a 31-year period, from 1989 to 2019, with particular attention to the first area of release in NW Italy. We collected data on species distribution through observations by citizen science projects, population density by transects with distance method, breeding censuses at colonies, and post breeding censuses at roosts. The birds counted at winter roosts in NW Italy increased from a few tens up to 10,880 individuals in 2019. Sacred ibises started breeding in 1989, with a single nest in north-western Italy. The number of breeders remained very low until 2006, when both overwintering and breeding sacred ibises started to increase exponentially and expand their range throughout northern Italy with isolated breeding cases in central Italy. In 2019, the number of nests had increased to 1249 nests in 31 colonies. In NW Italy, the density of foraging birds averaged 3.9 ind./km^2^ in winter and 1.5 ind./km^2^ in the breeding period, with a mean size of the foraging groups of 8.9 and 2.1 birds respectively. Direct field observations and species distribution models (SDM) showed that foraging habitats were mainly rice fields and wetlands. A SDM applied to the whole Italian peninsula plus Sardinia and Sicily showed that the variables best related to the SDM were land class (rice fields and wetlands), altitude, and the temperature seasonality. The areas favourable for species expansion encompass all the plains of Northern Italy, and several areas of Tuscany, Latium, Sardinia, and Apulia.

## Introduction

Biological invasions are considered one of the main factors related to the loss of biodiversity^1–3^. Autochthonous animal species may suffer from competition for trophic and territorial resources. New species may impact ecosystem services^4,5^, force the indigenous ones to shift their ecological niches or be confined in unfit areas, and trophic competition may limit food availability to the native species^6^. In some cases, native species can directly represent a source of nourishment for the invaders^7^. Another additional danger of the exotic species is their possible role as vectors of new pathogens, for which the indigenous species did not evolve resistance^8^. From a genetic point of view, the arrival of an invasive species would determine a loss of the native genotypes of different subspecies or even of the whole autochtonous species in case of hybridization^9,10^.

Nevertheless, the real danger posed by allochtonous invaders should not be presumed, instead it should be estimated for each case, different management options should be evaluated in relation to the arrival of a new species^11,12^, and alien *vs* alien and invasive species should be clearly differentiated^13^. One of the most accredited solutions is the eradication of the invading species^14^. However, when eradication involves killing, this poses ethical problems and might led to a block of the initiative^15^. Hence, conservation projects should distinguish allochthones that would cause negligible effects from those that cause significant damage to biodiversity. An invasive species can have a major impact in some areas and marginal effects in other sites^16^. Therefore, it is important to conduct appropriate ecological investigations on the alien species before starting any intervention^17–19^.

The sacred ibis *Threskiornis aethiopicus* is a wading bird native of Africa and the Middle East. The species is found in mudflats and wetlands, both on the coast and inland. It forages by wading in shallow waters or by trampling in moist grassland, and can build its nest in trees or on the ground^20,21^. This ibis was common in Egypt where it became extinct in 1850^22^. It is easily bred in many wildlife parks all over the world. In Europe, the sacred ibis has been released into the wild since the seventies. Several releases repeatedly occurred in Spain, Portugal, Germany, and the Netherlands^23^. Following the escape of individuals from these wildlife parks, wild breeding, range expansion and population increases have been observed in France^24^. Sporadic breeding occurred in several other Countries of western and central Europe, east Asia, and north America, where however no spreading over a vast area occurred. The two most important population explosions occurred in France from 1993 to 2000^24,25^, and after 2000 in Italy. The ability to adapt to new environmental conditions and the resulting increase of the escaped populations have included this ibis in the list of particularly invasive alien species^26^.

The first nesting in Italy occurred in a heronry at the Lame del Sesia park in Piedmont in 1989^27^. Since then, the population of Italian sacred ibis has increased considerably and has expanded its breeding range by colonizing several existing heronries in Piedmont and Lombardy. Some cases of nesting occurred also in northeastern Italy (Emilia-Romagna and Veneto) and in Tuscany. Wintering or moving individuals were recorded throughout all northern Italy and in Tuscany, Latium, and Sardinia. However, most of the Italian wild ibises are still confined to NW Italy. Sacred ibises in Europe are opportunistic feeders consuming a wide range of invertebrate and vertebrate prey^28^, particularly Amphibia^29^ and occasionally also autochthonous bird species^30^.

The first aim of this work was to describe the spread and the abundance of the sacred ibis in Italy since its appearance in 1989 up to 2019. We utilized: (a) incidental observations collected by voluntaries for citizen science projects; (b) density estimates calculated from transect lines with the distance-sampling method; (c) censuses at the breeding colonies; (d) winter censuses at roosts.

The second objective of our study was to build a species distribution model, using presence data, 19 bioclimatic variables^31^, altitude, and CORINE land use classes, in order to predict the areas more suitable for the spread of this invasive species.

## Methods

### Incidental observations

The sacred ibis, a medium size wader, is easily observed in nature for its unmistakable appearance, plumage predominantly white with contrasting black tips of remiges and tertials, naked black neck and head, large and curved bill. It is also clearly visible from a distance in its usually open habitats, so it is easily detected by nonprofessional birders. In this study we used a dataset composed of 10,260 records. We utilized the data collected by three projects of citizen science: 627 records from the AVES database of GPSO running in NW Italy from 1999^32^, 7717 records from the Ornitho.it of CISO running throughout all of Italy from 2009 (Lardelli R., pers. Comm.^33^), 733 records from the iNaturalist database by 2005^34^. We also used information collected in annual reports of national, regional, and local ornithological associations (459 records, respectively extracted from: EBN annual report, 147; Associazione Faunisti Veneti annual report, 176; Emilia-Romagna annual report, 6; GPSO annual report, 75; Checklist of birds of Modena, 21; Cronaca Ornitologica Toscana, 12; Liguria Birding, 22). We also added to the data set some records of occasional personal observation from us and other observers (55). We finally used 669 incidental observations collected by us during distance sampling.

### Distance sampling

In the period April 2017–February 2019, we utilized the distance sampling to assess the density of the species in NW Italy^35,36^. Two observers in a car travelled at low speed along road transects. Each time an ibis was detected, the observers stopped and measured the distance of the individual by a laser telemeter (Leica Rangemaster LRF 800 or LRF 900). In total we travelled along 2034 km (N = 140 transects with a mean of 14.5 km each). Transect data were grouped in two periods:

- *post breeding* from November to February (47 transects),
- *breeding* from April to August (93 transects).

We utilized the software Distance 7.2^37^ to calculate the density of the birds in our study area. We estimated abundance by taking into account the decreased probability of detection with increasing distance from the observer, using the tools provided by Distance, that compare different models of distribution (uniform, half normal, etc.). The best detection function retained was that with the smallest Aikake Information Criterion AIC^37^.

### Post-breeding census at roosts

In NW Italy, from the end of October, sacred ibises roost for the night in a few habitual sites, both colony sites of the previous breeding season or other suitable wood patches. We performed coordinated post-breeding censuses within a single week in November, from 2016 to 2019. The observers monitored each roost from a vantage point about 100 m distance, from 1 h before sunset to 30 min after the last sacred ibis had landed. The arriving birds could be easily counted as they arrived from the foraging areas in the open, cultivated landscape surrounding the roost. They usually fly towards the roost in groups of a few tens to some hundreds. The number of roosts checked each year varied between 13 and 19. As the sacred ibises tend to utilize the same location for several years, our census was repeated all along the study period in the first detected roost sites, while the remaining sites were added when they were discovered after the first year. In order to check for the presence of undetected roosts and to ensure the representativeness of the roosts we had monitored, we repeatedly inspected the landscape up to 15 km away from each site, during both the month before and after the post-breeding census.

### Census at breeding colonies

In Italy, sacred ibises always nested within colonies of other waterbirds, mostly heronries located in inland or coastal wetlands, in patches usually covered by alder or willow woods, bushes of *Salix* or *Tamarix* sp., or reedbeds, and less frequently in acacia groves and poplar plantations. These heronries were plurispecific, with one or more species of herons, egrets, and cormorants. In one case only, sacred ibises in NE Italy placed their nests on the ground among low bushes of *Salicornia* sp. within a colony of *Platalea leucorodia* and *Larus michahellis*.

The census of breeding sacred ibises' sites took advantage of the long-term initiative “Garzaie-Italia” that has been monitoring the heronries in Italy since 1972^38–40^. The active heronries monitored throughout northern Italy increased in number from 40 in 1972 to 301 in 2019^39,41^, and unpublished data in the “Garzaie-Italia” archive. The number of nests of each breeding species was estimated from ground observations and from aerial surveys by small drones, depending on the site characteristics and observability, during repeated visits to each colony from March to July, i.e. during the period of peak occupation by each species. According to the methods adopted for the other species that bred in the heronries, the counts of sacred ibis nests aimed to assess the “number of nests during the period of peak occupation” in order to obtain estimates that could be comparable among years. The counts included all visible nests without checking its contents. The count of sacred ibis nests presented additional difficulties, compared to the nest of the other waterbirds. Sacred ibis nests were often packed in communal platforms comprising from 2 to several nests, and their precise number was difficult to assess from the ground. Since 2017, an increasing number of cases of active nests were observed as late as September, but these nests were not included in the counts, because it could not be ascertained whether they were replacements of lost ones or new late breeding attempts. Several juvenile or adult individuals of sacred ibises were often observed roaming around the nest platforms, but they were not quantified, because they were apparently non-breeding. A precise count of sacred ibis nests could be performed only in 90% of cases, therefore we managed missing counts^42,43^ using the program TRIM (TRends and Indices in Monitoring data). TRIM allows for missing counts in the time series and produces unbiased yearly indices and standard errors using Poisson regression^44^. In this study, we excluded heronries data collected in the period from 1972 to 1989, because the first report of a nesting sacred ibis concerns a pair breeding in 1989^27^, while no other heronries were occupied by sacred ibises before 1989^39^.

To compare the species composition of heronries were the sacred ibis was present respect to heronries where the sacred ibis was absent, we analyzed the frequency of occurrence of waterbird species in colonies with sacred ibises against the frequency inside other colonies found within a 20-km radius but without them. The Yates chi-squared test was utilized to correct for continuity^45^.

### Habitat selection and species distribution model

We utilized the observations collected in citizen science projects^46^ to calculate species distribution models (SDMs).

We calculated two SDMs, the first concerning a detailed habitat analysis in NW Italy, i.e. the region with a highest occurrence recorded for the species, and a less detailed SDM concerning the whole Italian peninsula, Sicily, and Sardinia.

In NW-Italy, we utilized the detailed PTF cartography provided by Regione Piemonte administration^47^ to define habitat distribution in the study area. The PTF maps come from aerial photographs collected in 2016 and are converted into maps at the 1:10,000 scale. Habitats were originally codified in 126 land use classes. We clumped these classes into 17 classes of interest for the sacred ibis (Table 1). We also utilized the digital terrain model (DTM) provided by Italian Istituto Geografico Militare to calculate a topographical parameter, the altitude. DTM was downloaded at 5 × 5 m accuracy, then the GDAL connection plugin from QGIS software (GDAL/OGR contributors 2019) was utilized to calculate altitude at a 50 × 50 m grid size. In the detailed NW-Italy model we did not insert the bioclimatic variables.

**Table 1:** Land use classes utilized in the species distribution models.

**N**	Italy—land class	N	NW Italy—land class
1	Urban	1	Urban
2	Arable land	2	Arable land
3	Permanently irrigated	3	Non irrigated
4	Rice fields	4	Permanently irrigated
5	Heterogeneous agricultural	5	Rice fields
6	Vineyards, Fruits, Olive	6	Vineyard, Orchards
7	Pastures	7	Broad-lived
8	Broad-lived	8	Pastures
9	Mixed forests	9	Poplar
10	Coniferous	10	Acacia
11	Scrub	11	Coniferous
12	Grassland	12	Grassland
13	Open no vegetation	13	Scrub
14	Wetlands	14	Open no vegetation
15	Water	15	River beds
		16	Wetlands
		17	Water

For all of Italy land classes obtained from CORINE 2006 at 1:100,000 scale, and for NW Italy land classes obtained from PTF at 1:10,000 scale.

To build the SDM for the whole Italian peninsula plus Sardinia and Sicily, we utilized the CORINE cartography provided by ISPRA (CLC level III, http://www.sinanet.isprambiente.it/it/sia-ispra/download-mais) to define the habitat distribution in the area. The CORINE cartography is provided as maps at the 1:100,000 scale. Habitats were originally codified in 43 land use classes at level III. We clumped these classes into 15 classes of interest for the sacred ibis (Table 1). The DTM provided by Italian Istituto Geografico Militare was utilized to calculate the altitude. We utilized the dataset of 19 bioclimatic variables provided by the Worldclim organization^31^ (ver. 2.0) to compare the presence of the sacred ibis and the climatic conditions^48^. The 19 bioclimatic variables can be highly correlated, as well as the altitude, and this can cause inflation errors. To reduce the number of variables and select only uncorrelated ones, we utilized a variance inflation factor (VIF) approach^49^. We utilized the package *usdm* in R^50^ to select variables from the input variables that may have a collinearity problem. According to^51^, collinearity was avoided by removing the variables with VIF values > 10.

We utilized the software Maxent (version 3.3.3, https://biodiversityinformatics.amnh.org/open_source/maxent/) to calculate both the detailed model in NW Italy and the large SDM in all of Italy. Maximum entropy models have been found to be particularly robust for SDM estimations in cases where presence-only data is available^52,53^, and has been utilized in several studies of bird distribution^48,54^. In the Maxent model, we inserted the points with ascertained presence of sacred ibis, the land use classes as categorical variables, and the altitude and bioclim values as continuous variables. The program was run with these settings: remove duplicate presence records, random seed, regularization multiplier = 1,10,000 maximum number of background points, 500 maximum iterations, cross-validate replicated run type (80% of records were randomly extracted for training and 20% for testing the model). The predictive performance of the models was tested analyzing the receiver operated characteristics area under curve (AUC) and the true skill statistic (TSS)^55^. The relative importance of each predictor was assessed using the percent contribution to jackknife test.

Because sampling bias is frequently present in occurrence records^48^, we removed some of the bias by subsampling records. To reduce the overfit in oversampled areas, we rarefied the observations collected in nearby localities using the *gridsampler* package^56^ in R. We selected only one record for each cell in a grid 0.02 × 0.02 degrees (about 2.2 × 2.2 km). The records utilized in the model dropped from 10,260 to 1252 observations. A preliminary model run with 8884 records (selected by the "remove duplicate presence records" option in Maxent, without any grid sample technique) showed very similar results, in accord with the indication that changing grain size does not severely affect prediction for SDM^57^.

We also conducted a finer assessment of the habitat utilized by sacred ibis using field observations collected during line transects. Each time the observers stopped their transect trip to measure by laser the distance from the transect line of each detected individual, they also recorded the detailed habitat category: stubble or uncultivated land (STU); sown field (SOW); meadow (MEA); plowed field (PLO); farmhouse or isolated houses (FAR); inhabited center (INH); tree rows (ROW); poplar grove (POP); banks of the rivers or embankments of irrigation canals (EMB); dry rice fields (RIC); flooded rice fields (FLO); partially flooded rice fields (PAR).

## Results

### Incidental observations

**Figure 1:**
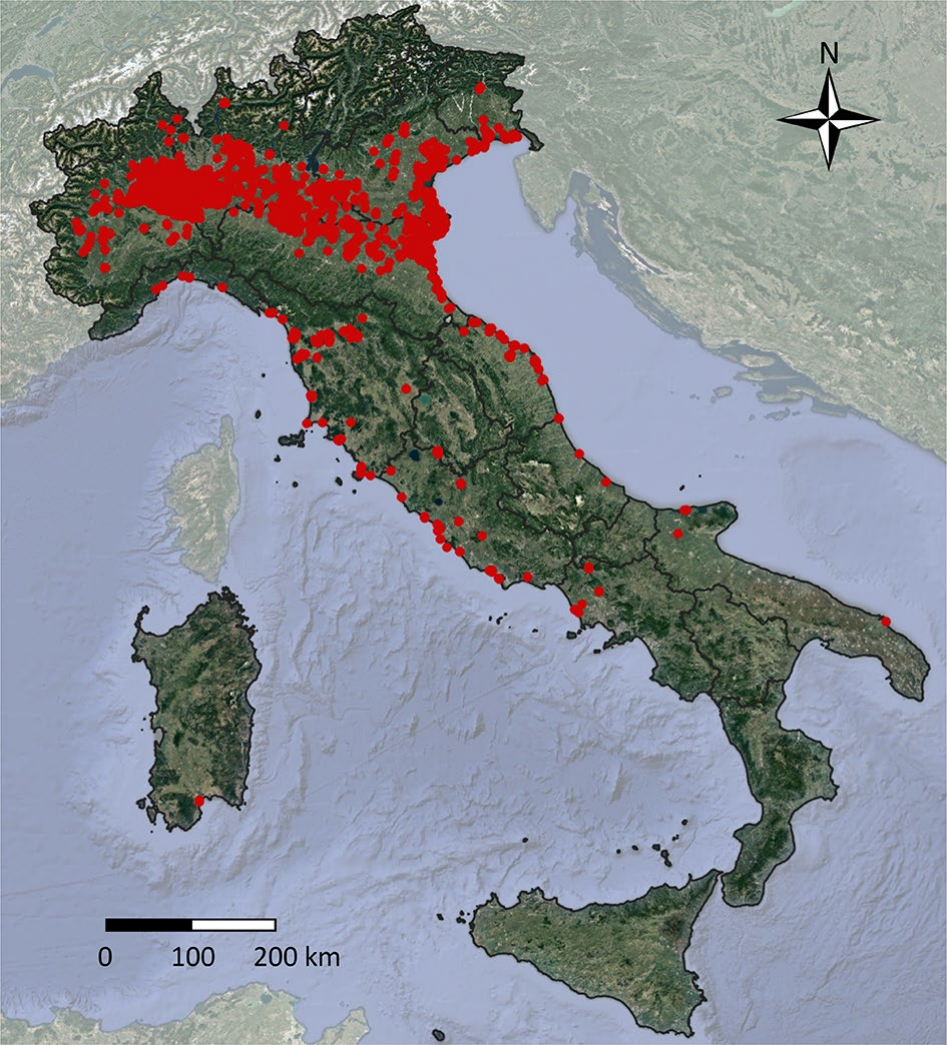
Incidental observations: distribution of the sacred ibis in Italy in the period from 1989–2019. Maps generated using QGIS ver. 3.4 (www.qgis.org).

The records of sacred ibises along the whole 1989–2019 study period in Italy are reported in Fig. 1. Detailed year-after-year distributions are reported in Supplementary Material 1. After the establishment of a first free living group near the breeding colony at Lame del Sesia (NW-Italy) in 1989, the observations occurred mainly within nearby areas until 1995, and in a few localities of Tuscany and Emilia Romagna until 2000. The main expansion in northern Italy occurred in 2010, when the species was abundantly recorded in a wide area on the Po plain, and in central Italy after 2015 when records became common in Tuscany, Marche, and Latium, and some individuals reached Apulia in southern Italy. From 2017, some individuals were observed in Sardinia.

### Transect distance sampling

**Figure 2:**
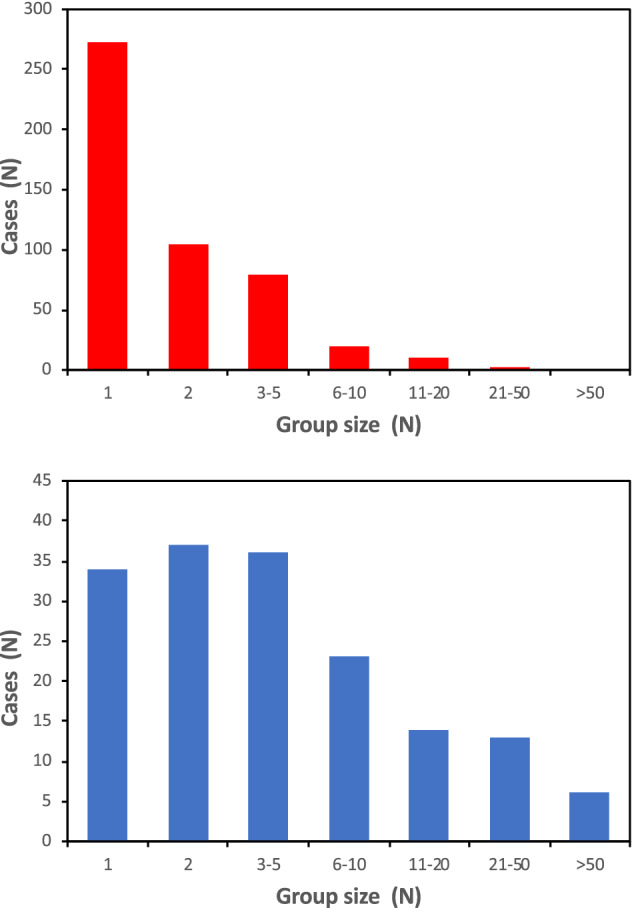
Distance sampling: group size in breeding (red) and winter (blue) periods.

During the daytime, sacred ibises disperse from their night-time roosts in order to forage in the surrounding areas. The density of individual sacred ibises, recorded in the Vercelli and Novara provinces of NW Italy from 2017 to 2019 (Supplementary Material 2), averaged 1.48 ± 0.29 ind./km^2^ during the breeding period, and 3.85 ± 0.94 ind./km^2^ during the winter season. The average group size was 2.08 ± 0.11 ind. during the breeding period, and 8.95 ± 1.27 ind. during the winter seasons (Fig. 2).

### Post breeding census at roosts

**Figure 3:**
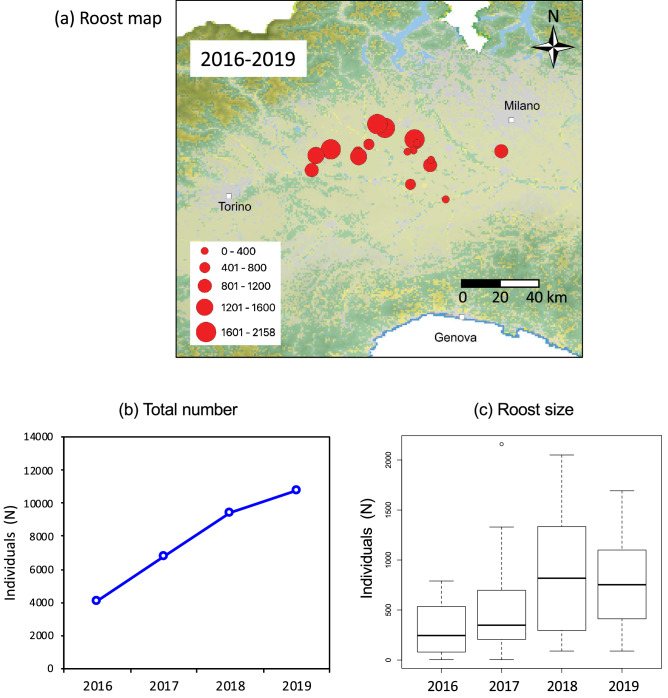
Sacred ibis presence at post-breeding roosts in the period from 2016–2019: (**a**) map of the roost sites in NW Italy; (**b**) total number of individuals; (**c**) median size of roosts. Maps generated using QGIS ver. 3.4 (www.qgis.org).

The censused roosts were located in the provinces of Vercelli, Novara, Alessandria, and Pavia (Fig. 3a). In the first census year we counted 4068 individuals within 13 roosts. The increase of ibises was continuous in the following years, with 6765, 9419, and 10,830 roosting individuals in 2017, 2018, and 2019 respectively (Fig. 3b). The roost size varied significantly during the study period (Kruskal–Wallis rank test = 8.307, d.f. = 3, P = 0.04), increasing from a median of 245 and 348 in the first 2 years to 716 and 753 in the last two (Fig. 3c). The maximum number of individuals in the largest roost was 790 in 2016, 2158 in 2017, 2050 in 2018, and 1692 in 2019.

### Census at breeding colonies

The first reports of free nesting sacred ibises occurred in 1989 and concerned one nest in a heronry in the Lame del Sesia natural park, Vercelli province, plus some nests in the Cornelle private park, Bergamo province (Supplementary Material 1). In 1998 at the Lame del Sesia natural park, 9 nests were estimated, 25 juveniles were observed fledging, and a maximum of 48 adults were counted. In the early 2000s, the only active breeding area remained in the Vercelli province, but trophic movements in the rice fields of the nearby province of Novara begun to be reported. Up to 2006, all the breeding sites were within NW Italy, only one or two breeding colonies existed, and the number of nests remained low. In 2006, the first colony was observed in NE Italy, where the number of occupied sites totalled four in recent years. Outside northern Italy, only a few attempts were recorded after 2016 in northern Tuscany.

**Figure 4:**
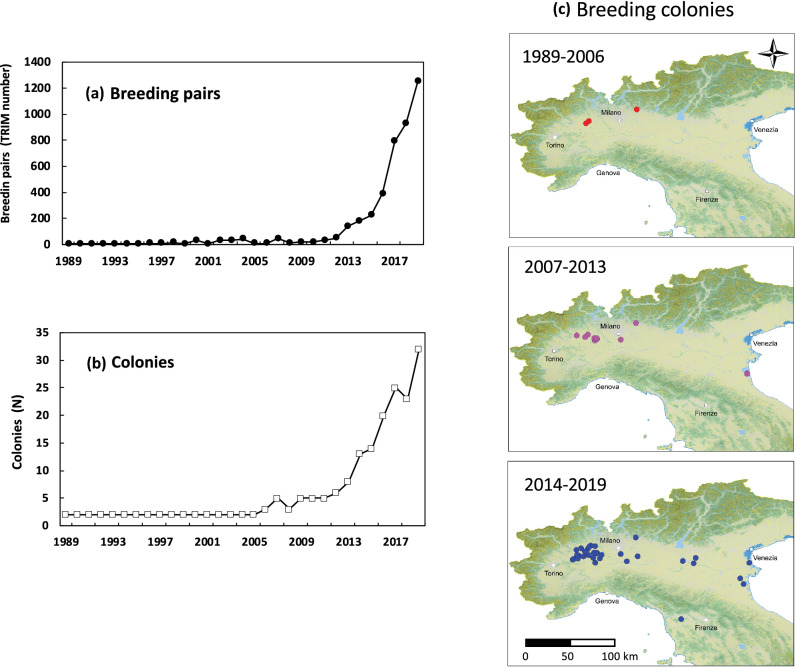
Sacred ibis presence at breeding colonies in the period from 1989–2019: (**a**) total number of pairs, TRIM estimates; (**b**) number of breeding colonies; (**c**) map of the breeding sites in Italy. Maps generated using QGIS ver, 3.4 (www.qgis.org).

Until 2011, the number of nests and colonies increased slowly, while after 2012, the number of both increased exponentially (Fig. 4). In 2019, sacred ibises bred at 31 colonies with a total of 1261 nests.

All breeding sacred ibises shared their colonies with other waterbirds. In NW Italy, where most colonies were located, the associated species were Grey Herons *Ardea cinerea* (present in 23% of the sacred ibis colonies), Purple Herons *Ardea purpurea* (5%), Cattle Egrets *Bubulcus ibis*, Little Egrets *Egretta garzetta*, Night Herons *N. nycticorax* (22% for each of the three species) and Cormorants *Phalacrocorax carbo* (6%). Others, less abundant species of colonial waterbirds (e.g. Glossy ibis *Plegadis falcinellus*) occurred in few cases. The frequency of occurrence of these waterbirds in colonies with sacred ibises did not differ significantly from their occurrence in the other colonies found within a 20-km radius but without them (Yates chi-square 5.06, df = 5, P = 0.41). The mixed colonies with sacred ibises in NW Italy were located on small tree patches (7% of cases), trees and bushes within inland wetlands (38%), riverine woodland vegetation (10%), dry woods (20%), reedbeds (3%), and trees in suburban parks or near inhabited landscapes (21%). Again, the frequencies of these habitats did not differ between colonies with or without ibises within 20-km (Yates chi-square 0.99, df = 5, P = 0.91).

### Species distribution models

In the detailed SDM computed for NW-Italy, we inserted the altitude and the land use class as predictors. The altitudes with a higher percent probability of presence were below 300 m above sea level, and there was a strong preference for a single land use class, the rice fields (Supplementary Material 3). All other land use classes showed a low predicted probability of presence. The eastern part of the region, where rice fields are widespread, was predicted to be the most favourable location for the presence of the species.

**Figure 5:**
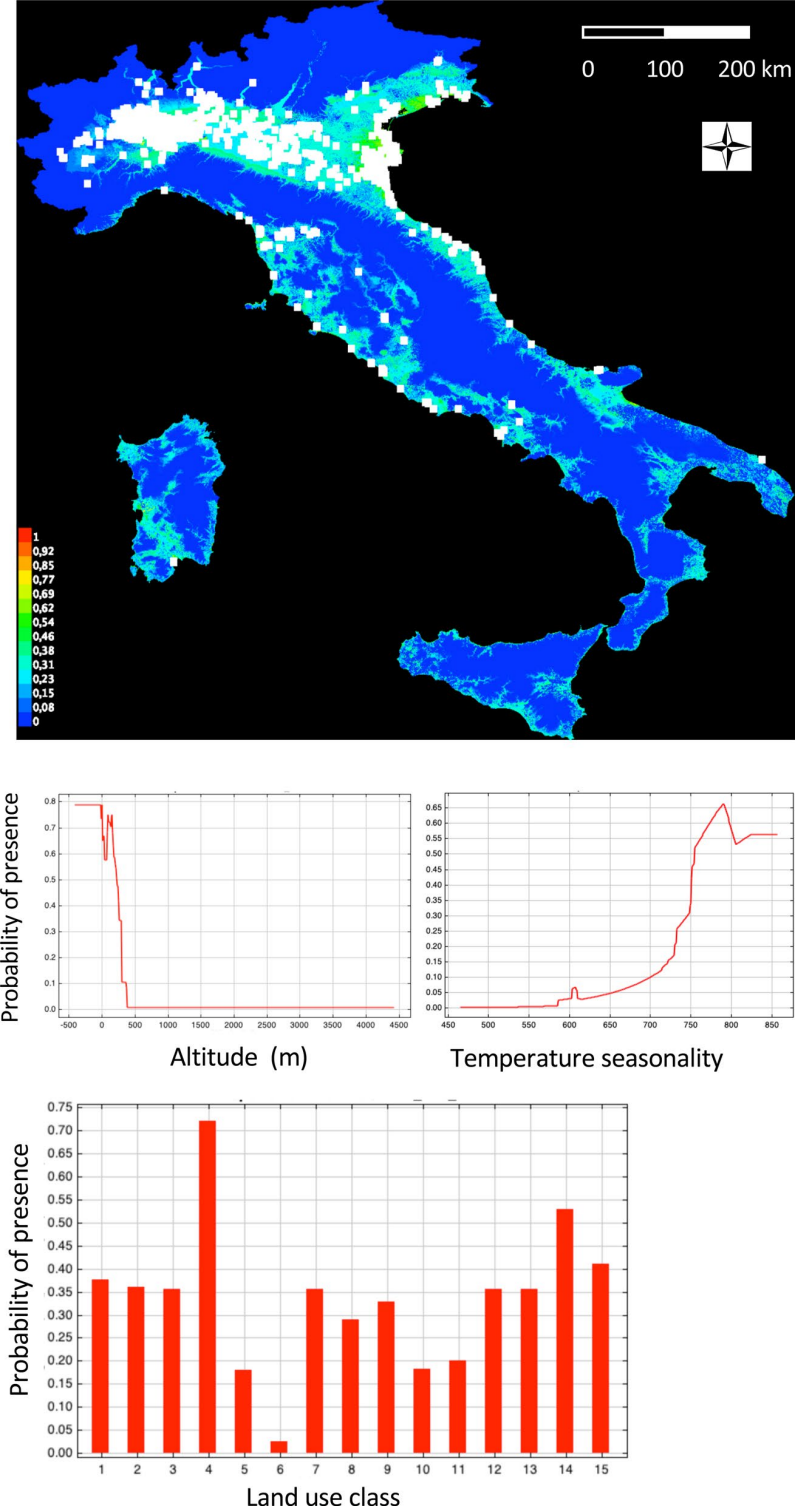
Species distribution model for the sacred ibis in Italy. Temperature seasonaly (Bio4 variable). Land use class names are reported in Table 1. Maps generated using QGIS ver, 3.4 (www.qgis.org).

The species distribution model computed for the whole Italy (the model had: area under curve AUC training data = 0.955; AUC test data = 0.943; true skill statistic TSS = 0.826) indicates a higher probability of presence below 300 m altitudes, and a selection for two land use classes: rice fields and wetlands. The other land use classes showed a low predicted probability of presence (Fig. 5). In Italy, all mountain or hilly areas of the Alps and Apennines were predicted to be unfavourable. All the northern Italy plains, from Piemonte in NW to Friuli and Veneto in NE, as well as several plains in central and southern Italy, a small part of Sicily, and a vast area of Sardinia were favourable for the presence of the species.

Results of the model which includes altitude, land use, and bioclimatic variables are reported in Supplementary Material 4. The VIF preliminary analysis of multicollinearity retained 9 out of 19 bioclimatic variables to be inserted in the SDM. The model confirmed the results of the previous model both for altitudes (below 300 m) and land use (higher probabilities in rice fields and wetlands). The bioclimatic variable that most influenced the probability of presence of the species was bioclim4 (temperature seasonality), with higher predicted suitability in high temperature seasonality areas. With respect to the previous model, this SDM predicted less favourable conditions for the presence of the species in Sicily, southern Italy, and most of Sardinia (Supplementary Material 4).

In the rice-field area of north-western Italy, where the sacred ibis is actually more abundant, a detailed assessment of the foraging habitats was performed during field transects. Most individuals were observed in rice fields, particularly in the boundary areas of the rice check (EMB: N = 194 cases), in the partially watered plots (PAR: N = 108), and in watered plots and rice fields (FLO and RIC: N = 76 and 32). Several observations were also performed in stubble or uncultivated land (STU = 86) and in ploughed fields (PLO = 86). The birds were rarely observed in sown fields and meadows (N = 51), did not perch on poplar groves or tree rows, and did not forage in the town interiors nor in their surrounding suburbs (0%).

## Discussion

Sacred ibises have been able to expand their presence in several areas outside their historical range, albeit with differing success. In this study, we show the pattern of spreading of this invasive species in Italy. There were only a few pairs of breeding sacred ibises from 1989 to 1997, which slowly increased over time. Only 9 pairs were recorded in 1998 with 25 fledglings and 24–26 pairs in 2000^27^. In the early 2000s, the only active breeding area was located in the northwestern Italy, in the Vercelli province, but trophic movements in the rice fields of the nearby Novara province were frequently reported. The first large group of ibises out of the Vercelli province was reported at Casalbeltrame, Novara, in 2006 (180 individuals on February 15). After 2006, the expansion of the sacred ibis was continuous and exponential. The number of breeding sites increased to 32 in northern Italy, with an estimated number of breeding pairs reaching 1249 in 2019. In winter, the birds aggregated at night in large roosts that recently reach a maximum of two thousand individuals. In 2019, the number of wintering ibises reached 11,000 in 19 roosts within NW Italy, and several other individuals were observed wintering in other areas of northern Italy. The recent increase in breeding sacred ibis in northern Italy mirrors the sudden increase observed in France during the 1994–2006 period. In France, 20 individuals were imported from Kenya and were sorted into different wildlife parks through four deliveries between 1975 and 1980. In a short time, a reproductive colony settled at the Branféré Zoological Garden in southern Brittany. In a natural habitat, the species was first noticed in 1993 near Golfe du Morbihan^24^ and Lac de Grand Lieu^58^. Then, the population of the Atlantic coast begins to increase up to 450 breeding pairs in 2001, while in 2005 there were already 1,100 pairs^24^. In France, the first winter census was carried out in the winter 2003–2004, with 2,500 ibises counted, while in the winter 2004–2005 there were about 3,000 individuals. In 2006, the number of reproductive pairs reached 1700 with a winter estimate now exceeding 5,000 individuals. In France other escapes occurred in the Mediterranean, but none was as successful as that of the Atlantic coast^59^.

In other parts of Europe, no such widespread diffusion and increase has occurred^60^. In Spain, since 1974, some individuals escaped from the Barcelona Zoo; in Portugal, there were some nesting attempts near the Coimbra park. Escapes from captivity occurred in the Netherlands, Great Britain, Germany, Belgium and Poland^23^. Outside the European continent, some sacred ibises were reported in Florida, where the species has been present since the mid-nineties with occasional breeding near the Miami Metro Zoo^61^. A small population has been regularly present on the island of Sir Bani Yas, in the United Arab Emirates, since 1989^25^. The species entered the wild in Taiwan before 1984 after a dispersal from a damaged zoo enclosure. Current estimates reach 2500 to 3000 birds, with an increase from 300 nests in 2016 to 800 nests in 2018^62,63^.

Our study shows that, after the first reports of nesting observed in northwestern Italy, the population remained at a low level until 2005, along a 17-year period. A sudden increase of breeding and roosting individuals occurred later, during the last 14 years. In all cases, the population expansion was carried out by colonizing existing heronries. Outside Piedmont, cases of nesting have been reported in Lombardy, Emilia-Romagna, Veneto, plus a temporary attempt in Tuscany. The increase of the breeding population was accompanied and preceded by a diffusion of the species in new areas. The observations collected by the citizen science projects in Italy show that the Italian population has recently expanded considerably. Reports of individuals or groups, in movement and/or wintering, were recorded in various areas of central Italy, and some individuals were observed in the southern peninsula (Apulia). From 2017, some individuals were observed in Sardinia, but it is unclear whether they reached the island from the continent or, more probably, they were due to a local escape of captive birds^64^.

Breeding outside the main northern Italy area is still isolated and occasional and does not affect the total national population count. However, they can play the role of a bridgehead in areas of post-reproductive dispersion and function as aggregation centres for prospecting immature subjects. Indeed, in the heronries of Ferrara, where the ibis recently nested, we observed up to some dozen immature individuals compared to the low number of adults (Volponi, pers. obs.).

Even if the species is present in several sectors of the Italian peninsula, large numbers are still limited to the northwest, including the Vercelli and Novara provinces. In this area, the density of foraging individuals was lower during the breeding period and increased to 3.85 ind./km^2^ during the winter season, when the juveniles that were born during the summer add up to the count. The animals forage alone or in small groups during the breeding period, while the group size increases noticeably in the winter seasons. The mean density reached in Italy is still lower than that found in the best areas of Ethiopia^65^, but sometimes birds can aggregate in nearby rice fields and reach high local densities in Italy too.

All our species distribution models clearly predicted a higher probability of presence of sacred ibises in rice fields and wetland areas^66^. All other land use classes were less likely to be utilized. This sharp habitat selection lent to a map of predicted areas for possible expansion of the species that covers all the regions of northern Italy, where rice cultivation is widespread, as well as the vast wetland area of the Po delta and the Venice and Grado lagoons in northern Italy. In central and southern Italy there is no tradition of rice cultivation, but the presence of the species is predicted where wetland areas do exist, particularly along the coasts. Part of the Sardinia island also represents an area of predicted high probability of presence of the species. Here, wide wetland areas and rice fields are present in the Oristano and Cagliari provinces, the last being the zone where the first observations of the species occurred^67^. The SDM including the bioclimatic variables indicated a lower probability of presence in southern Italy and Sicily, and a reduction of the area of presence in Sardinia. This model is strongly influenced by bioclim4 (temperature seasonality), with higher predicted presences in high temperature seasonality areas. Indeed, values of this climatic factor are high in the northern Po plain, an area that shows a continental climate, and lower in southern regions that show a Mediterranean climate.

In the future, the expansion of the sacred ibis in Italian rice areas could be limited by the recent modality of rice cultivation with limited flooding periods^68^. In the last two decades, the water presence in paddy fields is becoming more and more limited. This circumstance could negatively affect the possibility of foraging on water environments for both herons and egrets of conservation concern^68,69^, but for the invasive ibises too.

In the Atlantic coast of France, the foraging habitats comprised agricultural landscape, wet meadows, tidal habitats, and saltpans in the period 2000–2004, but then the ibises switched to rubbish dumps and freshwater marshes after the arrival of the invasive Red Swamp Crayfish *Procambarus clarkii*, which is an important element in their diet^60^. This crayfish is widespread in Italy^70,71^, but is common in rice fields and freshwater wetlands, the currently preferred environments for ibis, thus probably reducing the ability to promote a feeding habitat change in our study area.

The source of the Italian population is still unknown. It is not ascertained whether ibises originated from individuals who have escaped locally from zoological gardens, collectors, or breeders^72^, or if the Italian settlements have originated from individuals dispersing from the French populations. The first observations of free ranging sacred ibises in Italy were recorded in different regions (Piemonte 1998, Emilia 1991, Friuli 1988), without a geographic trend^73^. These data support the hypothesis of local escapes with respect to a progressive diffusion from western Italy near France towards the east. Future genetic studies^74^ could clarify whether Italian ibis populations pertain to different founder events, or are the descendants of a single original nucleus.

Currently, all breeding sites observed in Italy are multi-specific. During reproduction, the sacred ibis always mixes with herons or egrets in their heronries. This condition is different from that observed in western France during the sudden expansion of 2000–2005^60^, where the breeding colonies of ibis were monospecific. This circumstance has strong consequences in terms of management of the species, because any intervention on the nest site, the eggs, or the chicks of the ibis will cause a disturb that can negatively impact the associated herons, egrets, and other waterbirds, most of them currently deserving conservation concern^22^. Moreover, a number of heronries in Italy are now included in natural parks and in nature reserves, some of which have been designed specifically for the protection of waterbird colonies and are therefore submitted to strict regulation^75^. The continuous increase in the number of sacred ibises in Italy requires guidelines for the management of this alien species. In 2016, the sacred ibis was included in the list of invasive alien species (IAS) of Union relevance (EU Implementing Regulation 2016/1141), in application of EU Regulation 2014/1143 containing provisions aimed at preventing and managing the introduction and spread of invasive alien species. However, until recently, its presence was commonly advertised by zoos and they were offered for sale by exotic bird breeders located in various Italian regions, including Sardinia and Sicily^76^. Risk assessment and management options have been proposed in the Netherlands^77^, Belgium^23^, and France^60^. In Florida, scientists suggested that the most effective management strategy is eradication of the few pioneering individuals that are nesting, as well as the urban source population^61,78,79^.

Management of alien species is a difficult task. A strong debate exists about to what extent invasive species are dangerous for local species and cause their extinction^80–82^. Unfortunately, data on the impact of alien birds in most cases are lacking^83^. Among the risks posed by invasive species, hybridization should also be taken into account. In Europe, hybrids of sacred ibis have been sporadically reported with African Spoonbill and Scarlet ibis^84^, and there is no danger of large scale hybridization with taxonomically close species. In the case of eradication plans, public attitudes to the management of invasive non-native species should be carefully taken into account^85^. This study represents a snapshot of the current distribution of the sacred ibis in Italy, of the increase in nesting colonies and roosting sites, and predictions of new areas were future expansion will likely occur through the use of species distribution models. These data will help scientists and conservation ecologists to build risk analysis and management strategies for the species.

## Supplementary Information

Supplementary Information.

## Data Availability

Data are shown in Figures.
