# Supplementary material for: The spreading of the invasive sacred ibis in Italy
Source: Sci Rep. 2021 Jan 8;11:86. doi: 10.1038/s41598-020-79137-w (PMC7794294; doi:10.1038/s41598-020-79137-w)
Supplement: Supplementary file 1 — Supplementary Information. [file 41598_2020_79137_MOESM1_ESM.pdf]

The spreading of the invasive sacred ibis in Italy

Marco Cucco[1*], Gianfranco Alessandria[2], Marta Bissacco[1], Franco Carpegna[2], Mauro Fasola[3], Alessandra Gagliardi[4], Laura Gola[5], Stefano Volponi[6] & Irene Pellegrino[1]

[1]*University of Piemonte Orientale, DISIT, viale Michel 11, 15100 Alessandria*
[2]*GPSO, Museo di Storia Naturale, 12022 Carmagnola TO*
[3]*University of Pavia, DEES, via Ferrata 6 - 27100 Pavia*
[4]*Istituto OIKOS, via Crescenzago 1, 20134 Milano*
[5]*Parco del Po, piazza Giovanni XXIII 6, 15048 Valenza AL*
[6]*ISPRA, via Ca' Fornacetta 9, 40064 Ozzano Emilia BO*

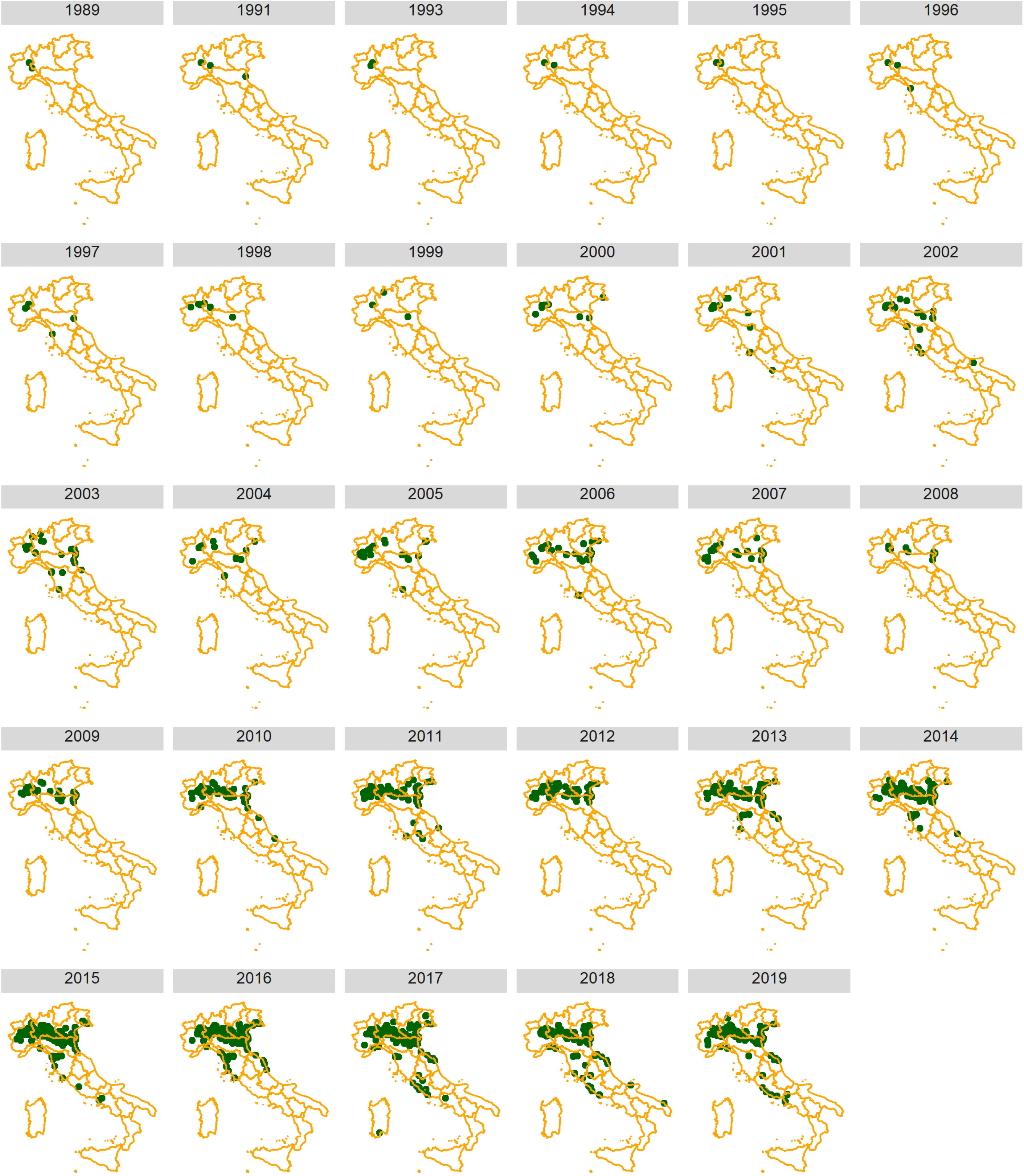

Supplemental material 1 - Incidental observations: detailed distribution of the Sacred ibis in Italy in each single year along the period 1989-2019.

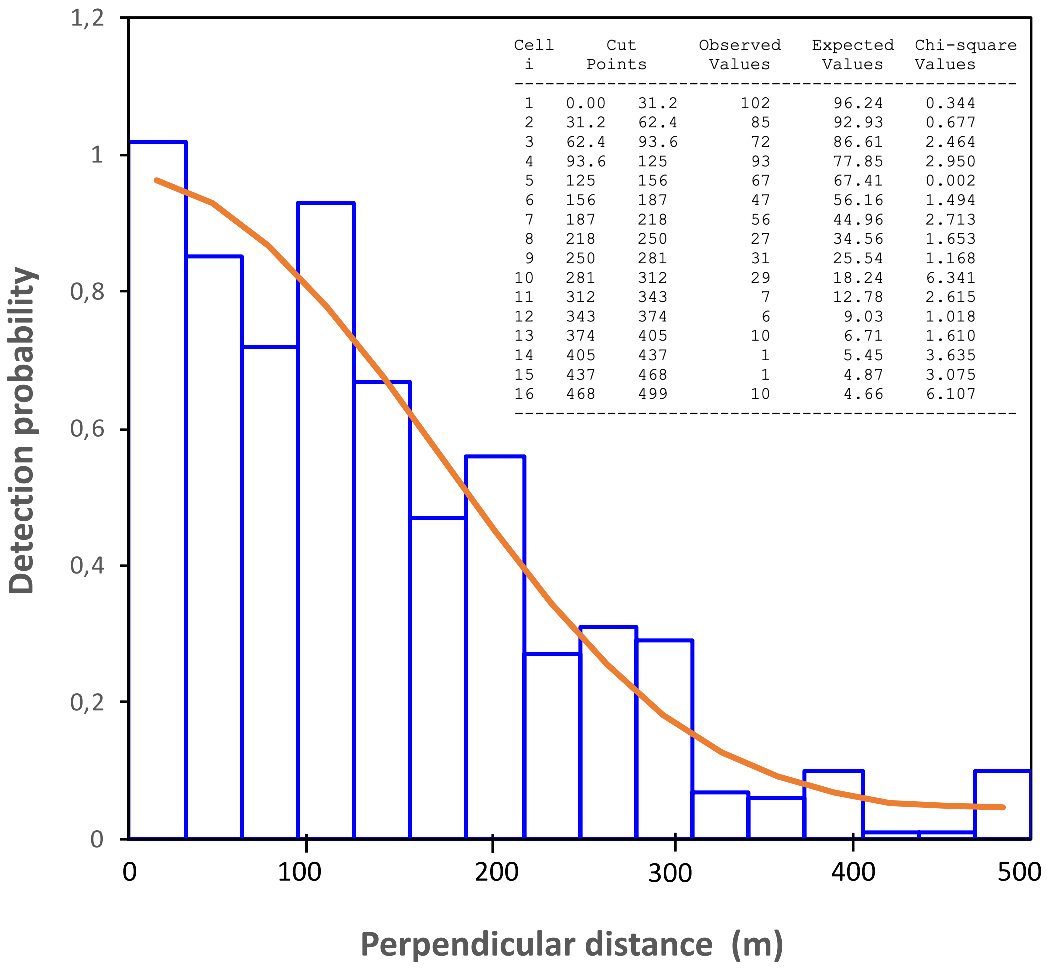

Supplemental material 2 - Detection probability along census transects in NW Italy.

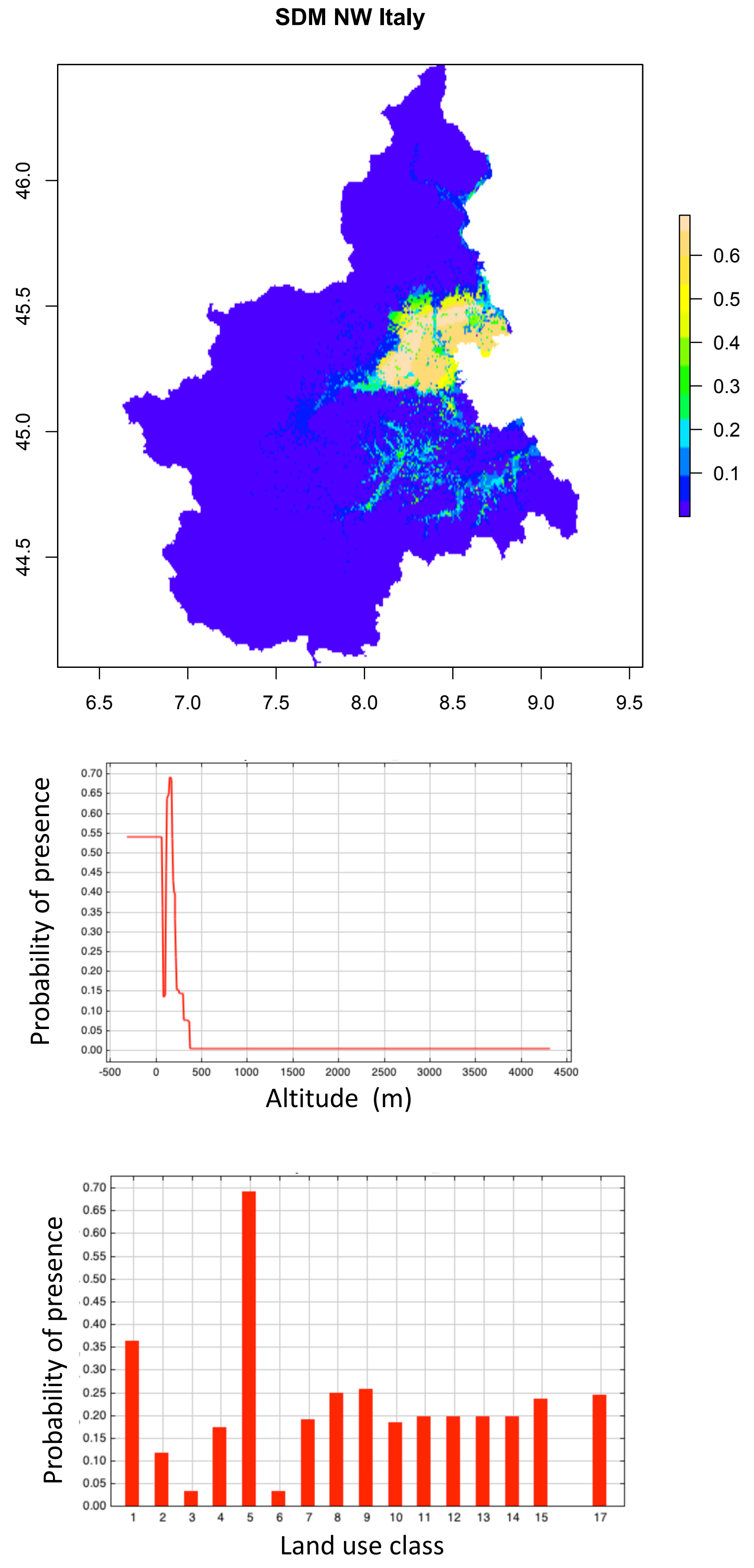

Supplemental material 3 - Detailed species distribution model in Piedmont, NW Italy. Land use class names are reported in Table 1.

Supplemental material 4 - Environmental variables utilized for modelling the distribution of the sacred ibis. Collinearity was avoided by removing the variables with inflation factor (VIF) values > 10.

**Variable**	**Code**	**VIF**	**VIF retained**
Annual mean temperature	Bio1	133.558488	
Mean diurnal range (max. Temp – min. temp)	Bio2	15.460259	
Isothermality (Bio2/Bio7) × 100	Bio3	13.025336	1.697651
Temperature seasonality (SD × 100)	Bio4	56.537905	3.214451
Max temperature of warmest month	Bio5	130.394299	
Min temperature of coldest month	Bio6	99.918143	7.600086
Temperature annual range (Bio5-Bio6)	Bio7	51.532417	4.522596
Mean temperature of wettest quarter	Bio8	5.486069	
Mean temperature of driest quarter	Bio9	7.503039	6.137563
Mean temperature of warmest quarter	Bio10	148.859108	
Mean temperature of coldest quarter	Bio11	148.368207	
Annual precipitation	Bio12	167.411067	
Precipitation of wettest month	Bio13	52.476940	9.832534
Precipitation of driest month	Bio14	41.577168	
Precipitation seasonality (CV)	Bio15	12.238587	3.164738
Precipitation of wettest quarter	Bio16	100.225181	
Precipitation of driest quarter	Bio17	69.613910	
Precipitation of warmest quarter	Bio18	34.839238	8.977698
Precipitation of coldest quarter	Bio19	25.771373	7.774096
